# Advancing sensory neuroprosthetics using artificial brain networks

**DOI:** 10.1016/j.patter.2021.100304

**Published:** 2021-07-09

**Authors:** David Haslacher, Khaled Nasr, Surjo R. Soekadar

**Affiliations:** 1 Clinical Neurotechnology Laboratory, Neurowissenschaftliches Forschungszentrum (NWFZ), Department of Psychiatry and Psychotherapy (CCM), Charité – Universitätsmedizin Berlin, Charitéplatz 1, 10117 Berlin, Germany

## Abstract

Implementation of effective brain or neural stimulation protocols for restoration of complex sensory perception, e.g., in the visual domain, is an unresolved challenge. By leveraging the capacity of deep learning to model the brain’s visual system, optic nerve stimulation patterns could be derived that are predictive of neural responses of higher-level cortical visual areas *in silico*. This novel approach could be generalized to optimize different types of neuroprosthetics or bidirectional brain-computer interfaces (BCIs).

## Teaser

Implementation of effective brain/neural stimulation protocols for restoration of complex sensory perception, e.g., in the visual domain, is an unresolved challenge. By leveraging the capacity of deep learning to model the brain’s visual system, optic nerve stimulation patterns could be derived that are predictive of neural responses of higher-level cortical visual areas *in silico*. This novel approach could be generalized to optimize different types of neuroprosthetics or bidirectional brain-computer interfaces (BCIs).

## Main text

While neuroprosthetics for restoration of movement have substantially advanced over the last years, implementing effective sensory neuroprosthetics proved very challenging because effective brain/neural stimulation protocols were lacking. In this issue of *Patterns*, Romeni et al. propose a method to optimize optic nerve stimulation parameters for vision restoration using an artificial brain network.^1^ By performing *in silico* experiments, they found that their stimulation framework achieves results comparable to natural vision. Such work highlights the potential of neurotechnology informed by artificial models of the brain and suggests that artificial neural networks may substantially aid the development of bidirectional brain-computer interfaces (BCIs) restoring both perception and action.

Neuroprosthetics, i.e., systems that substitute for motor, sensory, or cognitive functions, require neural interfaces that can interact with the brain. Such interaction builds on brain/neural signal *decoding*, e.g., to restore motor function in paralysis,^2^ and *stimulation* of neural tissue or nerves, e.g., for restoration of sensory function. Based on operant conditioning of neural cell assemblies and machine learning, motor and cognitive neuroprosthetics have achieved remarkable versatility, e.g., continuous control of individual finger, wrist, and hand movements using surface or implanted functional electric stimulation (FES).^3^^,^^4^ Recently, such a system was successfully enhanced by somatosensory cortex stimulation that substantially improved prosthetic hand and arm control by evoking tactile sensations.^5^ In another study, an implantable cortical interface was employed for high-performance brain-to-text communication via imagined handwriting long after motor function was lost.^6^ Besides restoration of motor and cognitive functions, neuroprosthetics have also successfully restored sensory function, e.g., in the auditory domain using cochlear implants.^7^ Similarly, simple vision could be restored using retinal implants,^8^ but restoration of more detailed visual perception was challenging due to the intricate spatiotemporal patterns of retinal or optic nerve activity that encode such perception. Building on the postulate that “every good regulator of a system must be a model of that system”,^9^ Romeni et al. (2021)^1^ created an artificial model of the visual system by capitalizing on a remarkable property of convolutional neural networks (CNNs). When such artificial brain networks are trained to classify images, the resulting model turns out to be highly predictive of neural responses of mid-level visual areas (e.g., V4) of the brain’s ventral stream.^10^ Importantly, this type of model can be pre-trained. Then, using the resulting rich artificial neuronal model of the visual system, inputs to the network could be optimized, representing electrical stimulation of the optic nerve that best activated an abstract cortical layer coding for the object whose sensory input was to be enhanced. Psychophysical data exhibiting large inter-subject and within-subject variabilities were obtained with healthy human volunteers. Here, the authors showed that the performance of their proposed framework was in general comparable to the healthy volunteers’ performance and even exceeded it in easy visual classification tasks.

While the approach is promising, a few key issues remain to be resolved. The presented technique must be subject to *in vivo* validation. Furthermore, optimization of stimulation parameters is currently performed on an image-by-image basis and thus cannot be performed in real time. In the future, a continuous mapping from video recordings to optic nerve stimulation parameters will be necessary for restoration of dynamic vision.

Regardless of these obstacles, it is conceivable that neuroprosthetics of the future will broadly use artificial brain networks to increase the scope of interaction with the human brain. In this context, however, implementation of bidirectional BCIs or neuroprosthetics that merge an artificial and biological cognitive system in a *hybrid mind* raises a number of neuroethical questions—some of which are still not fully charted.^11^

## References

[1] Romeni S., Zoccolan D., Micera S. (2021). A machine learning framework to optimize optic nerve electrical stimulation for vision restoration. Patterns.

[2] Soekadar S.R., Witkowski M., Gomez C., Opisso E., Medina J., Cortese M., Cempini M., Carrozza M.C., Cohen L.G., Birbaumer N., Vitiello N. (2016). Hybrid EEG/EOG-based brain/neural hand exoskeleton restores fully independent daily living activities after quadriplegia. Science Robotics.

[3] Bouton C.E., Shaikhouni A., Annetta N.V., Bockbrader M.A., Friedenberg D.A., Nielson D.M., Sharma G., Sederberg P.B., Glenn B.C., Mysiw W.J. (2016). Restoring cortical control of functional movement in a human with quadriplegia. Nature.

[4] Ajiboye A.B., Willett F.R., Young D.R., Memberg W.D., Murphy B.A., Miller J.P., Walter B.L., Sweet J.A., Hoyen H.A., Keith M.W. (2017). Restoration of reaching and grasping movements through brain-controlled muscle stimulation in a person with tetraplegia: a proof-of-concept demonstration. Lancet.

[5] Flesher S.N., Downey J.E., Weiss J.M., Hughes C.L., Herrera A.J., Tyler-Kabara E.C., Boninger M.L., Collinger J.L., Gaunt R.A. (2021). A brain-computer interface that evokes tactile sensations improves robotic arm control. Science.

[6] Willett F.R., Avansino D.T., Hochberg L.R., Henderson J.M., Shenoy K.V. (2021). High-performance brain-to-text communication via handwriting. Nature.

[7] Macherey O., Carlyon R.P. (2014). Cochlear implants. Curr. Biol..

[8] Zrenner E. (2002). Will retinal implants restore vision?. Science.

[9] Conant R.C., Ross Ashby W. (1970). Every good regulator of a system must be a model of that system. Int. J. Syst. Sci..

[10] Yamins D.L., Hong H., Cadieu C.F., Solomon E.A., Seibert D., DiCarlo J.J. (2014). Performance-optimized hierarchical models predict neural responses in higher visual cortex. Proc. Natl. Acad. Sci. USA.

[11] Soekadar S.R., Chandler J., Ienca M., Bublitz C. (2021). On the verge of the hybrid mind. Morals & Machines.

